# A Comparison of Appalachian and Non-Appalachian Kentucky DUI Offenders

**DOI:** 10.13023/jah.0103.02

**Published:** 2019-09-27

**Authors:** Megan F. Dickson, Megan Kissel, J. Matthew Webster

**Affiliations:** University of Kentucky; Kentucky Division of Behavioral Health; University of Kentucky

**Keywords:** Appalachia, drinking and driving, opioid epidemic, DUI offenders

## Abstract

**Purpose:**

To help fill this void in the literature, the current study uses a statewide sample to examine how Appalachian DUI offenders differ from non-Appalachian DUI offenders in a predominantly rural state.

**Methods:**

Assessment records for 11,640 Kentucky DUI offenders who completed an intervention in 2017 were examined. Appalachian DUI offenders were compared to non-Appalachian metro and non-metro DUI offenders. Demographic information, DUI violation details, DSM-5 substance use disorder criteria, and referral information were compared using ANCOVAs and logistic regression models.

**Results:**

More than one-fourth of the sample was convicted in an Appalachian county. Compared to non-Appalachian DUI offenders, Appalachian offenders were significantly older and more likely to have a prior DUI conviction, to meet DSM-5 criteria for a drug use disorder, and to drive drug-impaired. Referral and intervention compliance also varied across groups.

**Implications:**

Results suggest that Appalachian DUI offenders are more drug-involved and have increased risk of recidivism. Findings indicate a need for practitioners to consider the distinct needs of Appalachian DUI offenders during service delivery. Future research should explore alternative intervention methods for preventing continued impaired driving in Appalachia given limited treatment availability in the region.

## INTRODUCTION

In recent years, nonmedical opioid use has continuously increased in rural Appalachia,^1^ a region characterized by disproportionately high rates of poverty, chronic pain, lack of health insurance, and limited service availability.^1^,^2^ Although the ongoing opioid epidemic and continued increases in drug use prevalence in rural Appalachia have often been linked to a number of public health problems, such as high rates of injection drug use, overdose fatalities, and hepatitis C, recent literature has highlighted an overlooked impact of this epidemic: driving under the influence (DUI).^3^

Driving under the influence is a well-documented public health concern often linked to increased risk of traffic accidents^4^ and fatalities,^5^ and is one of the most frequently committed offenses in the U.S.^6^ However, rural communities appear to be disproportionately affected by DUI behaviors. Reports have not only indicated higher DUI arrest rates in rural communities compared to urban areas,^6^ but also a higher prevalence of fatal traffic accidents involving an alcohol-impaired driver^5^ and high rates of drugged driving among rural DUI offenders.^7^ Other studies have indicated that rural DUI offenders have greater drug problem severity^8^ and are at increased risk for substance-use disorders^8^,^9^ compared to their urban counterparts. However, it remains unclear if these characteristics extend into rural Appalachian DUI populations. Further, there are no studies specifically examining differences between Appalachian and non-Appalachian DUI offenders, although one recent study found that rural Appalachian DUI offenders have extensive mental health problems and criminal and drug-use histories.^3^

This limitation of the literature is noteworthy given the unique characteristics of the rural Appalachian region and the known substance-use service barriers experienced by those who live there.^1^,^2^,^10^ In an effort to better understand how the treatment needs of Appalachian DUI offenders vary compared to DUI offenders from other geographic regions, and how those needs can be met, the current study examines differences between Appalachian DUI offenders and non-Appalachian DUI offenders in a predominantly rural state using a statewide dataset. Specifically, this study compares DUI offenders’ demographic characteristics, DUI histories, substance-use problem severity, and current DUI offense characteristics, including involved substances, drug and alcohol testing, and court-mandated intervention information. Since all Appalachian Kentucky counties are part of rural Central Appalachia where drug use is highly prevalent,^1^ it was expected that Appalachian Kentucky DUI offenders in the current sample would have a higher rate of drug-involved DUIs, including opioid-related DUIs, with greater drug use problem severity than non-Appalachian DUI offenders.

## METHODS

Under Kentucky law (KRS 189A.040(1)(a)), Kentucky-licensed drivers convicted of a DUI are required to receive a substance-use assessment by a state certified DUI assessor. Assessment records are submitted to the Kentucky Department of Behavioral Health, Developmental and Intellectual Disabilities via an online system (as outlined in 908 KAR 1:310). Depending on the offenders’ substance-use problem severity, service availability, and a biopsychosocial interview, assessors refer them to a 20-hour education program (Prime for Lifeｮ) and/or a treatment program(s), including outpatient, intensive outpatient, or residential treatment.

Assessment records provide offenders’ demographic information, details from their current DUI violation, results from the Diagnostic and Statistical Manual (Fifth Edition; DSM-5) checklist for substance-use disorders, and intervention referral and completion information. Demographic information includes gender, age at the time of conviction, and DUI conviction history. For their current violation, offenders self-report the types of substances involved in the DUI arrest (alcohol, marijuana, opioids, sedatives, and/or other drugs) and are asked whether they were alcohol and/or drug tested. DSM-5 criteria are used to identify offenders who meet alcohol-use disorder criteria and/or drug-use disorder criteria and level of problem severity. Lastly, intervention referral information includes the level(s) of care (education and/or treatment) to which the offender is referred and whether they are compliant with the intervention requirements.

**Figure 1:**
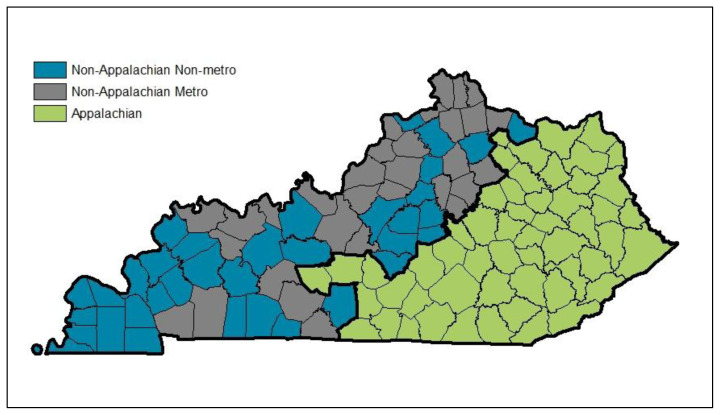
Appalachian and Non-Appalachian Counties

For the current study, the most recently available de-identified assessment records for offenders convicted of DUI in Kentucky were examined. The sample included 11,640 offenders who were assessed and completed a DUI intervention in 2017 (as either compliant or noncompliant), representing more than half (58.4%) of those convicted of DUI in Kentucky in 2017.^11^ As demonstrated in Figure 1, individuals were categorized into three groups based on whether they were convicted in an Appalachian county (n=3168), a non-Appalachian metro county (n=5779), or a non-Appalachian nonmetro county (n=2693). Appalachian counties are those which have been designated by the Appalachian Regional Commission as Appalachian. Non-Appalachian counties were classified as metro or nonmetro counties using Beale Codes from the U.S. Department of Agriculture Economic Research Service, which are assigned based on a county’s population and its proximity to a metropolitan area. For this study, non-Appalachian counties with Beale codes 1 through 3 were combined into a metro county category, while codes 4 through 9 were combined into a nonmetro category.

Appalachian DUI offenders were compared to non-Appalachian metro and non-Appalachian nonmetro DUI offenders using a series of ANCOVA and logistic regression analyses controlling for demographic differences. Using Appalachian DUI offenders as the reference group, analyses specifically examined differences in demographic information, offenders’ DUI substance involvement and measurement, DSM-5 criteria, and referral information. Analyses were conducted using SPSS 24.

## RESULTS

Overall, participants were mostly male (73.5%), with an average age of 36.1 years (SD = 12.6) at the time of their current DUI conviction. One fourth (25.9%) of the sample self-reported having a prior DUI conviction, 32.2% reported being drug-impaired at the time of their current DUI, and 27.2% were convicted in an Appalachian county. As presented in Table 1, analyses highlighted a number of significant differences between Appalachian and non-Appalachian DUI offenders. First, analyses indicated that Appalachian DUI offenders were older than non-Appalachian offenders (F(2,11,637)=17.1, p<0.001). Further, non-Appalachian metro (OR = 0.73, p<0.001, CI(95) = 0.66, 0.80) and nonmetro (OR = 0.89, p=0.05, CI(95) = 0.80, 1.00) offenders were less likely to self-report having a prior DUI conviction compared to Appalachian offenders.

Appalachian DUI offenders also indicated greater drug problem severity at the time of their assessment compared to non-Appalachian DUI offenders. Specifically, when controlling for age at conviction and prior DUI offense history, both non-Appalachian metro (AOR = 0.47, p<0.001, CI(95) = 0.42, 0.52) and non-Appalachian nonmetro (AOR = 0.81, p<0.001, CI(95) = 0.72, 0.91) offenders were significantly less likely to meet DSM-5 criteria for a drug use disorder. Compared to Appalachian DUI offenders, non-Appalachian metro (AOR = 0.73, p<0.001, CI(95) = 0.65, 0.82) offenders were also less likely to meet DSM-5 criteria for a severe substance-use disorder. Conversely, non-Appalachian metro (AOR = 3.23, p<0.001, CI(95) = 2.91, 3.58) and nonmetro (AOR = 2.09, p<0.001, CI(95) = 1.85, 2.35) offenders were more likely to meet DSM-5 criteria for an alcohol-use disorder.

**Figure 2:**
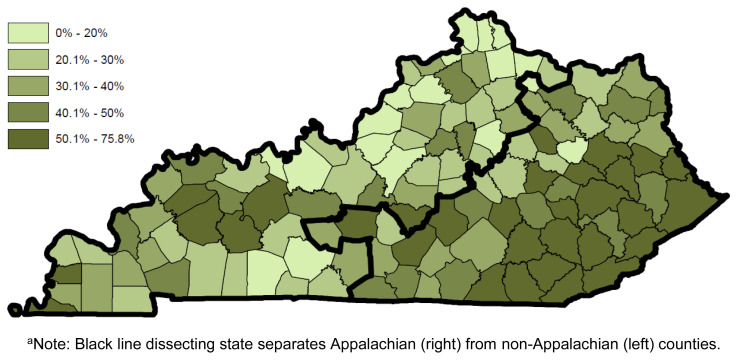
Prevalence of Drug-involved DUIs by County of Conviction (N=11,640) ^a^Note: Black line dissecting state separates Appalachian (right) from non-Appalachian (left) counties.

Substance involvement and substance testing also varied across groups. Compared to Appalachian DUI offenders, non-Appalachian metro (AOR = 0.26, p<0.001, CI(95) = 0.23, 0.28) and nonmetro (AOR = 0.56, p<0.001, CI(95) = 0.50, 0.62) offenders were significantly less likely to self-report being under the influence of drugs at the time of their current DUI. Figure 2 shows the prevalence of self-reported drug-involved DUI convictions for each county in Kentucky. Both non-Appalachian metro and nonmetro offenders were specifically less likely to report being under the influence of opioids (metro AOR = 0.24, p<0.001, CI(95) = 0.21, 0.28 and nonmetro AOR = 0.36, p<0.001, CI(95) = 0.30, 0.42); sedatives (metro AOR = 0.29, p<0.001, CI(95) = 0.24, 0.35 and nonmetro AOR = 0.53, p<0.001, CI(95) = 0.43, 0.66); and other drugs (metro AOR = 0.36, p<0.001, CI(95) = 0.31, 0.42; nonmetro AOR = 0.78, p=0.001, CI(95) = 0.67, 0.91), while only non-Appalachian metro offenders were less likely to report being under the influence of marijuana (AOR = 0.47, p<0.001, CI(95) = 0.41, 0.53). In addition, non-Appalachian metro (AOR = 0.29, p<0.001, CI(95) = 0.26, 0.33) and non-Appalachian nonmetro (AOR = 0.78, p<0.001, CI(95) = 0.69, 0.87) offenders were significantly less likely to self-report being drug tested.

On the other hand, non-Appalachian DUI offenders, both metro and nonmetro, were significantly more likely to self-report that their current DUI involved alcohol (metro AOR = 4.53, p<0.001, CI(95) = 4.09, 5.01 and nonmetro AOR = 1.91, p<0.001, CI(95) = 1.71, 2.13) and to report being tested for alcohol impairment (metro AOR = 1.81, p<0.001, CI(95) = 1.66, 1.97 and nonmetro AOR = 1.40, p<0.001, CI(95) = 1.26, 1.55).

Finally, data from offenders’ assessment records indicated that when controlling for age at conviction and prior DUI offense history, there were several differences in recommended interventions. Although the prevalence of education referrals did not differ across groups, non-Appalachian metro (AOR = 1.12, p=0.014, CI(95) = 1.02, 1.23) and nonmetro (AOR = 1.14, p=0.017, CI(95) = 1.02, 1.27) DUI offenders were significantly more likely than Appalachian offenders to be referred to outpatient treatment, while non-Appalachian nonmetro offenders were less likely to be referred to a more intensive treatment (intensive outpatient or residential; AOR = 0.66, p=0.004, CI(95) = 0.50, 0.88). Further, non-Appalachian metro offenders were less likely to be compliant with the intervention to which they were referred (AOR = 0.85, p=0.009, CI(95) = 0.76, 0.96).

## IMPLICATIONS

The present study examined similarities and differences between Appalachian and non-Appalachian DUI offenders in Kentucky. Despite a growing body of literature exploring rural DUI, the unique characteristics of rural Appalachia, and evidence of higher DUI conviction rates in Appalachian counties compared to non-Appalachian counties (5.7 vs. 4.0 per 1000 residents, respectively),^11^ few studies have specifically examined Appalachian DUI offenders. Overall, results suggest that Appalachian DUI offenders are older and more drug-involved than their non-Appalachian counterparts. Appalachian DUI offenders are also more likely to drive under the influence of drugs, including opioids, which is consistent with prior research highlighting the increasing rates of drug use in the Appalachian region.^1^ Given the well-documented challenges in accessing substance-use treatment services in rural Appalachia,^1^,^2^,^10^ increased drug use problem severity and high rates of drug-impaired driving signal important implications for Appalachian DUI offenders, such as improving treatment availability and accessibility.

Evidence of more severe drug use problems and known difficulties accessing substance-use treatment in rural Appalachia may explain the heightened recidivism risk among this sample of Appalachian DUI offenders compared to non-Appalachian DUI offenders. The potential risks posed by repeat DUI offenders, such as greater likelihood of being involved in a fatal motor-vehicle accident,^12^ elevate concerns surrounding access to substance-use treatment services in the rural Appalachian region. Given these barriers, it may be necessary to identify alternative intervention methods focused on preventing and reducing future drug-impaired driving among Appalachian DUI offenders. This need is further underscored by the rate of referral to more intensive forms of treatment. Despite one fifth of Appalachian DUI offenders meeting DSM-5 criteria for a severe substance-use disorder and 29.5% having a prior DUI conviction, less than half were referred to outpatient treatment and fewer than 5% were referred to either an intensive outpatient or residential treatment program. With almost a third of the Appalachian DUI offenders in the current sample having prior criminal justice involvement due to past DUI offenses, future research should examine the criminal justice system as an opportunity to assess and treat the substance-use treatment needs of Appalachian DUI offenders, as suggested with other rural populations.^2^

Contrary to previous studies which found rural DUI offenders to be less compliant^8^ with their recommended intervention, Appalachian DUI offenders in the present study were more likely to comply than non-Appalachian metro DUI offenders. This significantly higher rate of compliance could be explained in part by the lack of available employment opportunities^1^ in the Appalachian region and the potentially limited obligations interfering with the recommended intervention. The higher compliance rate could also be a function of the type of intervention to which offenders were referred. While not presented as part of study results, additional analyses indicated that offenders referred to outpatient treatment were the least likely to comply with referral recommendations across all groups. The significantly lower rate of referral to outpatient treatment in the Appalachian region, possibly due to service availability,^2^ could explain compliance rate differences.

Finally, results also point to an increased likelihood of sedative- and opioid-impaired driving among Appalachian DUI offenders compared to non-Appalachian offenders. Provided past research highlighting above-average misuse of prescription drugs in rural Appalachia as a characteristic of the ongoing opioid epidemic^1^ and evidence of high rates of sedative and prescription opioid use among another sample of Appalachian DUI offenders,^3^ study findings indicate a need for Appalachian DUI intervention programs to educate on the dangers of driving under the influence of prescription medications. Study results also suggest that future researchers should examine other, less recognized consequences of the opioid epidemic, such as DUI.

The current study has limitations that should be considered. First, data were collected in a single, predominantly rural state in which the Appalachian region is economically depressed relative to other Appalachian areas.^1^ This may affect the generalizability of study results. Future studies should continue to examine the characteristics of all Appalachian DUI offenders, including those in other, less rural areas of Appalachia. In addition, data were collected by multiple assessors and are largely self-report, which may affect data accuracy. However, certified DUI assessors in Kentucky are required to successfully complete a 3-day, in-person training prior to receiving their certification, helping to ensure similar assessment practices across the state. Finally, data for the current study are limited to convicted DUI offenders who received an assessment. Although past research has regularly utilized samples of convicted DUI offenders, other studies have highlighted discrepancies between the frequency of self-reported substance-impaired driving, DUI arrest, and conviction.^7^ Given that the likelihood of official arrest, conviction, and assessment could vary across counties as a result of factors such as police presence, future studies consider potentially confounding community-level variables when conducting similar research.

Despite these limitations, the current study fills an important gap in the literature by examining a largely understudied group of DUI offenders in a region significantly affected by the opioid epidemic. Study findings suggest Appalachian DUI offenders may have unique needs compared to non-Appalachian DUI offenders. Despite being more drug-involved and having more severe drug use problems, limited treatment availbility^1^,^2^ could result in higher recidivism rates among Appalachian DUI offenders. Future research should continue to examine this group of DUI offenders and explore alternative intervention methods for preventing continued drug-impaired driving in rural Appalachia, while further exploring less-recognized consequences of the opioid epidemic.

**SUMMARY BOX**

**What is already known about this topic?** Existing studies have found that rural DUI offenders have greater drug problem severity and are at increased risk for substance-use disorders, while rural Appalachian DUI offenders have specifically been shown to have extensive mental health problems and criminal and drug use histories.

**What is added by this report?** No existing studies have drawn direct comparisons between Appalachian DUI offenders and their non-Appalachian counterparts to determine if Appalachian DUI offenders have unique treatment needs. The current study provides important insight into the characteristics of Appalachian DUI offenders, who are more drug-involved, with greater substance-use problem severity, and at increased risk of recidivating compared to non-Appalachian DUI offenders.

**What are the implications for public health practice, policy, and research?** Findings suggest that practitioners should be sensitive to the distinct needs of Appalachian DUI offenders during service delivery, while future research continues to examine Appalachian DUI offenders and explore alternative intervention methods for preventing continued impaired driving in rural Appalachia.

## Figures and Tables

**TABLE 1:** Comparison of Appalachian vs. Non-Appalachian DUI Offenders in Kentucky (N=11,640)

	Appalachian^a^ (n=3168)	Non-Appalachian Metro (n=5779)	Non-Appalachian Nonmetro (n=2693)	Kentucky (N=11,640)
**Demographics**				
Age at conviction (SD)	37.2 (12.5)	35.6 (12.7)***	35.9 (12.7)***	36.1 (12.6)
Male (%)	74.0	72.7	74.4	73.5
Prior DUI conviction(s) in lifetime (%)	29.5	23.3***	27.1*	25.9
**DSM-5 Substance-use Disorder (%)**				
Drug-use disorder	29.2	16.8***	25.8***	22.3
Alcohol-use disorder	20.5	43.0***	33.8***	34.7
Severe substance-use disorder	20.0	15.0***	18.4	17.1
**Substance Involvement/Testing (%)**				
Drug-involved	49.4	21.0***	36.1***	32.2
Marijuana	16.8	9.8***	17.1	13.4
Opioids	19.3	5.3***	7.9***	9.7
Sedatives	9.4	2.8***	5.2***	5.2
Other Drugs	15.2	6.1***	12.3***	10.0
Alcohol-involved	55.9	84.4***	69.9***	73.3
Drug tested	31.5	12.2***	26.7***	20.8
Alcohol tested	47.2	61.6***	55.3***	56.2
**Highest Level of Care Recommended (%)**				
Education	51.5	51.8	50.7	51.5
Outpatient	44.2	44.7*	46.4*	44.9
IOP/Residential	4.3	3.5	2.9**	3.6
**Compliant (%)**	83.8	82.4**	82.5	82.8%

* *p*<0.05;

** *p*<0.01;

*** *p*<0.001

a Appalachian is the reference category for study analyses.
